# Finding of extended-spectrum beta-lactamase (ESBL)-producing Enterobacterales in wild game meat originating from several European countries: predominance of *Moellerella wisconsensis* producing CTX-M-1, November 2021

**DOI:** 10.2807/1560-7917.ES.2022.27.49.2200343

**Published:** 2022-12-08

**Authors:** Magdalena Nüesch-Inderbinen, Silvan Tresch, Katrin Zurfluh, Nicole Cernela, Michael Biggel, Roger Stephan

**Affiliations:** 1 Institute for Food Safety and Hygiene, Vetsuisse Faculty, University of Zurich, Switzerland

**Keywords:** Game meat, antimicrobial resistance, ESBL, *E. coli*, *Escherichia marmotae*, *Moellerella wisconsensis*

## Abstract

**Introduction:**

Meat can be a vehicle for food-borne transmission of antimicrobial resistant bacteria and antimicrobial resistance genes. The occurrence of extended‐spectrum beta‐lactamase (ESBL) producing Enterobacterales has been observed in meat from livestock production but has not been well studied in meat from wild game.

**Aim:**

We aimed to investigate, particularly in central Europe, to what extent ESBL-producing Enterobacterales may be present in wild game meat.

**Methods:**

A total of 111 samples of different types of game meat supplied by butchers, hunters, retail stores and a large game-processing establishment in Europe were screened for ESBL-producing Enterobacterales using a selective culture medium. Isolates were genotypically and phenotypically characterised.

**Results:**

Thirty-nine samples (35% of the total) yielded ESBL-producing Enterobacterales, with most (35/39) supplied by the game-processing establishment. Isolates included 32 *Moellerella wisconsensis*, 18 *Escherichia coli* and one *Escherichia marmotae*. PCR screening identified *bla*_CTX-M-1_ (n = 31), *bla*_CTX-M-32_ (n = 8), *bla*_CTX-M-65_ (n = 4), *bla*_CTX-M-15_ (n = 3), *bla*_CTX-M-8_ (n = 1), *bla*_CTX-M-14_ (n = 1), *bla*_CTX-M-55_ (n = 1), and *bla*_SHV-12_ (n = 2). Most *E. coli* belonged to phylogenetic group A (n = 7) or B1 (n = 9), but several isolates belonged to extraintestinal pathogenic *E. coli* (ExPEC) sequence types (ST)58 (n = 4), ST68 (n = 1) and ST540 (n = 1). Whole genome sequencing of six selected isolates localised *bla*_CTX-M-1_ on megaplasmids in four *M. wisconsensis* and *bla*_CTX-M-32_ on IncN_1 plasmids in one *M. wisconsensis* and one *E. marmotae*. Forty-eight isolates (94%) exhibited a multidrug-resistance phenotype.

**Conclusion:**

We found a high occurrence of ESBL-producing Enterobacterales in wild game meat, suggesting wildlife habitat pollution and possible microbial contamination events occurring during skinning or cutting carcasses.

**Key public health message**

**What did you want to address in this study?**

Wild game meat consumption is gaining popularity in many countries, but the prevalence of antibiotic-resistant bacteria in this type of meat it is not well known. In November 2021, we collected wild game meat samples from various European suppliers (butchers, hunters, retail stores and a game-meat-processing plant) to investigate for the presence of such bacteria.

**What have we learnt from this study?**

Wild game meat may contain antimicrobial resistant germs, including bacteria producing enzymes called extended-spectrum beta-lactamases (ESBLs). ESBL-producing bacteria are resistant to many beta-lactam antibiotics, such as third-generation cephalosporins which are used to treat a variety of infections, for example pneumonia and meningitis.

**What are the implications of your findings for public health?**

The potential transfer of ESBL-producing bacteria to humans constitutes a public health risk, as such bacteria may transmit their antimicrobial-resistance genes to pathogenic bacteria within the human gut, making infections harder to treat.

## Introduction

Bacterial resistance to antimicrobials is one of the most imminent threats to global public health [1]. Enterobacterales that produce extended-spectrum beta-lactamase (ESBLs) are of particular concern, since they are resistant to many beta-lactam antibiotics including extended-spectrum penicillins, monobactams and third generation cephalosporins, all of which are categorised by the World Health Organization (WHO) as critically important antimicrobials for human medicine [2,3]. Since the late 1990s there has been a rapid global emergence and dissemination of *Escherichia coli* and other Enterobacterales producing CTX-M type ESBLs within the healthcare setting, in livestock, and in food of animal origin [2].

During 2019–2020, specific monitoring of retail livestock meat at European Union (EU)-level showed that on average 23.4% of the broiler meat samples, 5.5% of the pork samples, and 4.3% of the beef samples contained ESBL-producing *E. coli* [4]. Throughout 2019 and 2020, in the EU and in Switzerland, monitoring of ESBL-producing *E. coli* in these meat categories was mandatory, but not in hunted wild game meat [4,5]. Wild game meat is gaining popularity in many countries and appeals to a growing demand for foods that are both nutritious and represent an alternative to meat from intensive livestock production [6]. Despite the growing importance of wild game meat, there are few reports addressing antimicrobial resistance (AMR) in this food category and most studies that report the possibility of human exposure to ESBL-producing Enterobacterales and transmissible *bla*_ESBL_ genes via the consumption of meat are focused on meat from domestic food-producing animals and do not include wild game meat [7]. Therefore, this study was designed to assess the prevalence of ESBL-producing Enterobacterales in wild game meat collected during November 2021.

## Methods

### Sample collection

The study comprised the analysis of 111 wild game meat samples collected during November 2021. The collection included 38 meat samples of red deer (*Cervus elaphus*), 42 of roe deer (*Capreolus capreolus*), 28 of wild boar (*Sus scrofa*), two of chamois (*Rupicapra rupicapra*) and one of European fallow deer (*Dama dama*).

The meat samples (which excluded offal) were either supplied by Swiss hunters from animals shot during the hunting season of 2021 (n = 21) or purchased at Swiss butcher shops (n = 22) and retail stores (n = 18). In addition, 50 meat samples were obtained from a large game meat processing establishment located in Slovenia. The establishment processes domestic and imported hunted game animals and produces game meat and meat products for the European market, including for Switzerland.

Overall, for the 111 game meat samples, the countries of origin included Austria (n = 9), Croatia (n = 1), Germany (n = 10), Hungary (n = 7), New Zealand (n = 1), Poland (n = 16), Slovenia (n = 22) and Switzerland (n = 45). An overview of the countries of origin and the suppliers of the game meat samples is given in Table 1.

**Table 1:** Country of origin and type of supplier of wild game meat samples, November 2021 (n = 111)

Country of origin	Supplier			
	Butcher n = 22	Hunter n = 21	Processing plant n = 50	Retail store n = 18
**Austria n = 9**				
Red deer	0	0	4	3
Wild boar	0	0	0	2
**Croatia n = 1**				
Red deer	0	0	1	0
**Germany n = 10**				
Red deer	0	0	0	5
Roe deer	0	0	0	3
Wild boar	0	0	0	2
**Hungary n = 7**				
Red deer	0	0	7	0
**New Zealand n = 1**				
Red deer	0	0	0	1
**Poland n = 16**				
Red deer	0	0	6	0
Roe deer	0	0	6	0
Wild boar	0	0	4	0
**Slovenia n = 22**				
Chamois	0	0	1	0
Red deer	0	0	6	0
Roe deer	0	0	10	0
Wild boar	0	0	5	0
**Switzerland n = 45**				
Chamois	0	1	0	0
Fallow deer	1	0	0	0
Red deer	3	1	0	1
Roe deer	13	9	0	1
Wild boar	5	10	0	0

### Screening for extended-spectrum beta-lactamase-producing *Enterobacterales*

Of each meat sample, 10–20 g were placed in a sterile blender bag (Seward, Worthing, United Kingdom (UK)), homogenised at a 1:10 ratio in Enterobacteriaceae enrichment (EE) broth (BD, Franklin Lakes, United States), and incubated at 37 °C for 24 hours.

For the detection of ESBL-producing Enterobacterales, one loopful of each of the EE cultures was streaked onto Brilliance ESBL agar plates (Oxoid, Hampshire, UK). Plates were incubated under aerobic conditions at 37 °C for 24 hours. Colonies with different coloration and growth morphology were sub-cultured on Brilliance ESBL agar plates at 37 °C for 24 hours. From each plate, single colonies were picked and sub-cultured on plate count agar (PCA) for 24 hours at 37 °C.

Species were identified using matrix assisted laser desorption ionization-time of flight mass spectrometry (MALDI-TOF-MS, Bruker Daltonics, Bremen, Germany). Bacterial identification was carried out using the software Flex Control 3.4., the MALDI Biotyper (MBT) Compass database version 4.1.100, and the MBT Compass Library Revision H 2021.

### Detection of *bla*_ESBL_ genes

Bacterial species found, which included *E. coli*, *E. marmotae* and *Moellerella wisconsensis* were characterised by identification of *bla* genes. Bacterial DNA was extracted using a standard heat lysis protocol. Screening for *bla*_TEM_ and *bla*_SHV_ was carried out using primers described previously [8]. Screening for *bla*_CTX-M_ alleles belonging to CTX-M groups 1, 2, 8, 9, and 25 was performed as described by Woodford et al. [9]. Amplicons for sequencing *bla*_CTX-M_ genes were generated using primers described previously [10]. Synthesis of primers and DNA custom sequencing was carried out by Microsynth (Balgach, Switzerland). Nucleotide sequences were analysed with CLC Main Workbench 21.0.4 (Qiagen, Aarhus A/S). For database searches the nucleotide–nucleotide Basic Local Alignment Search Tool (BLASTN) programme of the United States (US) National Center for Biotechnology Information (NCBI; http://www.ncbi.nlm.nih.gov/blast/) was used.

### Antimicrobial susceptibility testing

Antimicrobial susceptibility testing (AST) was conducted on the isolates using the disk-diffusion method according to the guidelines of the Clinical and Laboratory Standards Institute (CLSI) [11]. Antimicrobial substances included ampicillin, amoxicillin/clavulanic acid, cefazolin, cefotaxime, cefepime, nalidixic acid, ciprofloxacin, sulfamethoxazole-trimethoprim, fosfomycin, azithromycin, nitrofurantoin, streptomycin, kanamycin, gentamicin, chloramphenicol, and tetracycline (Becton, Dickinson, Heidelberg, Germany). Results were interpreted according to CLSI breakpoints for human clinical isolates [11]. Multidrug resistance (MDR) was defined as resistance to three or more classes of antimicrobials, counting beta-lactams as one class.

### Extended-spectrum beta-lactamase-producing *Escherichia coli* phylogenetic analysis and typing

The distribution of phylogenetic groups among the ESBL-producing *E. coli* was determined by PCR targeting the genes *chuA, yjaA, arpA* and TspE4.C2, as described by Clermont et al. [12]. Isolates were assigned to one of the following eight phylogenetic groups including the seven (A, B1, B2, C, D, E, F) belonging to *E. coli* sensu stricto, and one (the eighth), which is the *Escherichia* clade I.

For multilocus sequence typing (MLST) of *E. coli* isolates, internal fragments of the seven housekeeping genes (*adk, fumC, gyrB, icd, mdh, purA*, and *recA*) were amplified by PCR as described by Wirth et al. [13]. The amplification products were custom sequenced and sequence types (STs) were determined using the *E. coli* MLST database website (https://enterobase.warwick.ac.uk).

### Whole genome sequencing of *Moellerella wisconsensis* and *Escherichia marmotae*

Whole-genome sequences of six isolates were determined using short-read (Illumina MiniSeq) and long-read sequencing (MinION, Oxford Nanopore Technologies). The isolates were selected based on the following criteria; they (i) were constituted of Enterobacterales species for which data on genomic location of *bla*_ESBL_ genes are scarce (*E. marmotae* and *M. wisconsensis*), (ii) produced different ESBLs (CTX-M-1 or CTX-M-32), and (iii) were of relatively diverse origins, such as from the Croatian, Hungarian and Polish game meat samples obtained via the Slovenian processing plant or the Swiss sample obtained from a hunter. The isolates were grown on sheep blood agar at 37 °C overnight before DNA isolation. For short-read sequencing, genomic DNA was extracted using the DNA blood and tissue kit (Qiagen, Hombrechtikon, Switzerland). The DNA libraries were prepared using a Nextera DNA Flex Sample Preparation Kit (Illumina, San Diego, CA, US), and sequencing was done on an Illumina MiniSeq (Illumina, San Diego, CA, US). For long-read sequencing, genomic DNA was extracted using the MasterPure Complete DNA and RNA Purification Kit (Lucigen LubioScience, Zürich, Switzerland). Multiplex libraries were prepared using the SQK-LSK109 ligation sequencing kit with the EXP-NBD104 native barcoding expansion kit (ONT, Oxford, UK) and sequenced on a MinION Mk1B device using the FLO-MIN106 (R9) flow cell (ONT). Base calling, demultiplexing, and barcode trimming was performed with Guppy v4.2.2 (Oxford Nanopore Technologies, Oxford, UK) and quality assessed with LongQC v1.2.0 [14].

The 2×150 bp paired end Illumina-reads were trimmed with fastp 0.20.1 [15] and passed the standard quality checks using the software package FastQC 0.11.7 (Babraham Bioinformatics, Cambridge, UK). Hybrid assemblies were obtained using Unicycler v0.4.8 [16] with default settings.

The isolates’ genomes were then analysed for the presence of *bla*_ESBL_-encoding plasmids. Resistance genes and plasmid replicons were identified using abricate 1.0.1 (coverage/identity > 70%/ > 90%) [17] with the ResFinder [18] and PlasmidFinder [19] database, respectively. Similar plasmids were identified by querying the bacterial plasmid database PLSDB, available at https://ccb-microbe.cs.uni-saarland.de/plsdb/ [20] with a mash-distance of 0.01 as cut-off. The similarity to identified hits was determined by aligning the plasmids using BLASTN.

### Statistical analysis

Comparisons of proportions of samples containing ESBL-producing Enterobacterales from meat of different animal species, from different countries of origin of the meat samples, and different meat suppliers (butchers, hunters, production plant or retail stores) were performed by Fisher's exact test. For each comparison, the proportion of meat containing ESBL-producing bacteria in one item was compared to the proportion of ESBL-producers in all other items. The significance criterion was set at p ≤ 0.05. Calculations were performed using GraphPad (https://www.graphpad.com).

## Results

### Extended-spectrum beta-lactamase-producing Enterobacterales’ distribution among game meat samples

An overview of the number of samples collected per animal species, country of origin, and supplier is given in Table 2. Overall, 39 (35%) of the 111 game meat samples yielded ESBL-producing Enterobacterales. They included 24 of the 38 samples from red deer, nine of the 42 samples from roe deer, and six of the 28 samples from wild boar. The proportion of samples containing ESBL-producing Enterobacterales was significantly higher among meat from red deer and from roe deer than from other types of meat (Table 2).

**Table 2:** Wild game meat samples originating from several countries and from different suppliers, November 2021 (n = 111)

Characteristics	Proportion of meat samples containing ESBL-producing Enterobacterales among number of meat samples collected	p value^a^	Enterobacterales (number of isolates)
**Game meat type**			
Chamois	0/2	0.5400	NA (NA)
Fallow deer	0/1	1.0000	NA (NA)
Red deer	24/38	**< 0.0001**	*E. coli* (10), *E. marmotae* (1), *M. wisconsensis* (17)
Roe deer	9/42	**0.0240**	*E. coli* (3), *M. wisconsensis* (9)
Wild boar	6/28	0.1089	*E. coli* (5), *M. wisconsensis* (6)
**Country of origin**			
Austria	6/9	0.0641	*E. coli* (6)
Croatia	1/1	0.3514	*E. coli* (1), *M. wisconsensis* (1)
Germany	0/10	**0.0138**	NA
Hungary	5/7	0.0507	*E. coli* (1), *M. wisconsensis* (5)
New Zealand	0/1	1.0000	NA
Poland	11/16	**0.0039**	*E. coli* (10), *E. marmotae* (1), *M. wisconsensis* (4)
Slovenia	15/22	**0.0008**	*E. coli* (6), *M. wisconsensis* (15)
Switzerland	1/45	**< 0.0001**	*M. wisconsensis* (1)
**Supplier**			
Butcher	0/22	**< 0.0001**	NA (NA)
Hunter	1/21	**0.0008**	*M. wisconsensis* (1)
Production plant	35/50	**< 0.0001**	*E. coli* (15), *E. marmotae* (1), *M. wisconsensis* (31)
Retailer	3/18	0.1050	*E. coli* (3)

*E. coli*: *Escherichia coli*; *E. marmotae*: *Escherichia marmotae*; *M. wisconsensis*: *Moellerella wisconsensis*; NA: not applicable.

^a^ Fisher's exact test: p ≤ 0.05 are indicated in bold.

The 39 contaminated meat samples included six of the nine samples from Austria, the single sample from Croatia, five of the seven samples from Hungary, 11 of the 16 samples from Poland, 15 of the 22 from Slovenia, and one of the 45 samples from Switzerland. Proportions of samples with ESBL-producing Enterobacterales from Poland and Slovenia were significantly higher than those from other countries (Table 2).

The ESBL-producing Enterobacterales were detected in one of the 21 samples supplied by hunters, in 35 of the 50 samples originating from the game meat processing establishment and in three of the 18 samples purchased in retail stores. The proportion of meat samples containing ESBL-producing Enterobacterales was significantly higher among samples originating from the production plant than among samples from other suppliers (Table 2).

**Figure 1:**
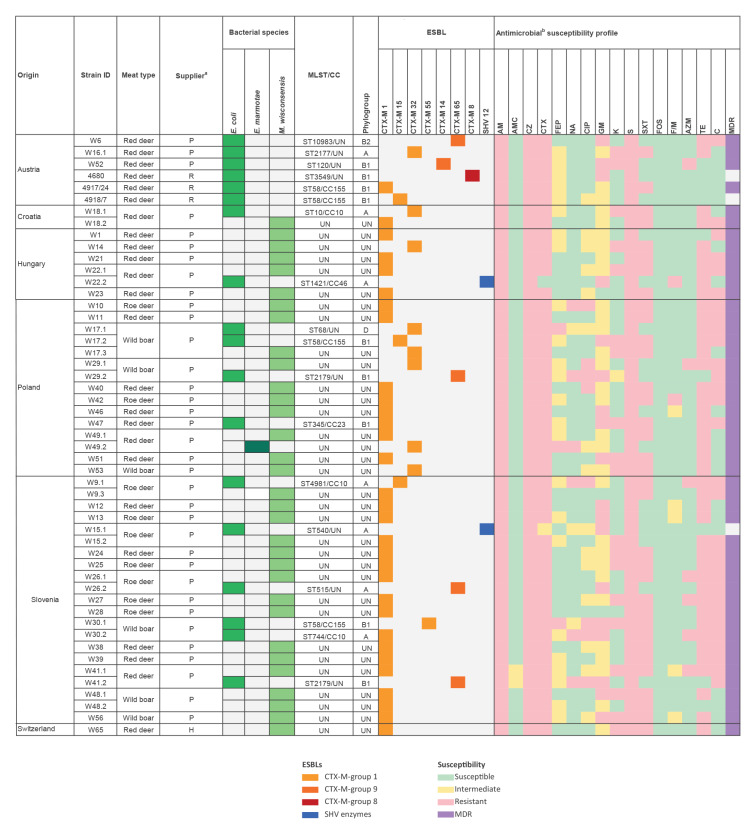
Source data, identity, distribution of *E. coli* sequence types and clonal complexes, *bla* genes and antibiotic susceptibility profiles of ESBL-producing Enterobacterales isolated from 39 wild game meat samples in different European countries, November 2021 (n = 51 isolates)

Of the 39 contaminated meat samples, 11 contained multiple isolates (two or three distinct ESBL-producing Enterobacterales). In total, 51 ESBL-producing isolates were retrieved. Of these, 32 (63%) were identified as *M. wisconsensis*, 18 (35%) were *E. coli* and one (2%) was *E. marmotae* (Figure 1).

CC: clonal complex; *E. coli*: *Escherichia coli*; *E. marmotae*: *Escherichia marmotae*; ESBL: extended-spectrum beta-lactamase; MLST: multilocus sequence type; *M. wisconsensis*: *Moellerella wisconsensis*; ST: sequence type; UN: unapplicable.

^a^ H: hunter; P: game meat processing establishment; R: retail store.

^b^ AM: ampicillin; AMC: amoxicillin-clavulanic acid; C: chloramphenicol; CIP: ciprofloxacin; CTX: cefotaxime; CZ: cefazolin; FEP: cefepime; F/M: nitrofurantoin; FOS: fosfomycin; GM: gentamicin; K: kanamycin; MDR: multidrug-resistant; NA: nalidixic acid; S: streptomycin; SXT: sulfamethoxazole-trimethoprim; TE: tetracycline; TMP: trimethoprim.

### Identification of *bla*_ESBL_ genes

All 51 isolates were characterised with respect to their ESBL genotypes. Of the 32 *M. wisconsensis,* 28 harboured *bla*_CTX-M-1_ and four carried *bla*_CTX-M-32_. Among the 18 *E. coli*, four carried *bla*_CTX-M-65_, three harboured *bla*_CTX-M-1_, three *bla*_CTX-M-15_, and three *bla*_CTX-M-32_, and one each carried *bla*_CTX-M-8_, *bla*_CTX-M-14_, and *bla*_CTX-M-55._ Two *E. coli* tested positive for *bla*_SHV-12_. The *E. marmotae* isolate harboured *bla*_CTX-M-32_ (Figure 1).

### Antimicrobial susceptibility phenotypes

Phenotypic AST revealed that all 51 isolates were resistant to ampicillin and to the first generation cephalosporin cefazolin. Further, 49 (96%) exhibited resistance to the third generation cephalosporin cefotaxime. Resistance to other categories of antimicrobials was common, with all 51 isolates resistant to streptomycin, 42 (82%) to tetracycline, and 34 (67%) to sulfamethoxazole-trimethoprim. Moreover, 48 (94%) of all isolates and 15 of the 18 *E. coli* isolates were MDR (Figure 1).

### Phylogenetic analysis of extended-spectrum beta-lactamase-producing *Escherichia coli*

Phylogenetic typing allocated 16 of the 18 *E. coli* isolates to group A or B1, which typically contain commensal *E. coli* strains. One isolate each (1/18) belonged to extraintestinal pathogenic phylogroup B2 and D, respectively (Figure 1). A total of 14 different STs were identified by MLST. Thereof, only two STs occurred more than once: four *E. coli* ST58 were found in two red deer samples from Austria and two wild boar samples respectively from Poland and Slovenia; two *E. coli* ST2179 were identified in one red deer sample from Slovenia and one wild boar sample from Poland (Figure 1). Of note, ST58 represents an extraintestinal pathogenic *E. coli* (ExPEC), as do ST68 and ST540, which were found in one wild boar sample from Poland and one roe deer sample from Slovenia in the present study (Figure 1).

### Extended-spectrum beta-lactamase-producing *Moellerella wisconsensis* and *Escherichia marmotae*

Genomes of five *M. wisconsensis* (four producing CTX-M-1 and one producing CTX-M-32) isolated from meat from different countries including Croatia, Hungary, Poland, and Switzerland were completely resolved to determine the genomic location (plasmid or chromosome) of the *bla*_CTX-M_. In addition, the CTX-M-1-producing *E. marmotae* was included for whole genome sequencing analysis. ESBL-encoding plasmids were identified in each isolate (Table 3). In four of the five *M. wisconsensis* isolates, *bla*_CTX-M-1_ was identified on megaplasmids (pW18–2-a, pW51-a, pW1-a, and pW65-a; ca 270–350 kb) which contained six to nine additional antimicrobial resistance genes (Table 3) and were largely homologous (67–88% alignment coverage, > 99% sequence identity).

**Table 3:** Characteristics of *bla*_ESBL_-carrying plasmids identified in *Moellerella wisconsensis* and *Escherichia marmotae* isolated from wild game meat samples from different European countries, November 2021 (n = 6 plasmids)

Isolate	Bacterial species	Meat type	Country of origin	Plasmid	Plasmid size (kb)	Inc type (pMLST alleles)	*bla*_ESBL_ gene	Additional antimicrobial resistance genes	Accession number
W18–2	*M. wisconsensis*	Red deer	Croatia	pW18–2-a	271	ni	*bla*_CTX-M-1_	*ant(3”)-Ia*, *aph(3”)-Ib*, *aph(6)-Id*, *floR*, *mph*(A) *lnu*(F), *sul2*	GCA_022591895.1
W51	*M. wisconsensis*	Red deer	Poland	pW51-a	271	ni	*bla*_CTX-M-1_	*aac(3)-IIa*, *aph(3')-Ia*, *aph(3”)-Ib*, *aph(6)-Id*, Δ*ant(3”)-Ia*, *dfrA1*, *mph*(A), *sul2*, *tet(*D)	GCA_022592015.1
W1	*M. wisconsensis*	Red deer	Hungary	pW1-a	332	ni	*bla*_CTX-M-1_	*aph(3”)-Ib*, *aph(6)-Id*, *floR*, *lnu*(G), *mph*(A), *sul2*	GCA_022592275.1
W65	*M. wisconsensis*	Red deer	Switzerland	pW65-a	345	ni	*bla*_CTX-M-1_	*aph(3')-Ia, *Δ*ant(3”)-Ia*, *dfrA1*, *mph*(A), *sul3*, *tet(*H)	GCA_022592395.1
W17–3	*M. wisconsensis*	Wild boar	Poland	pW17–3-a	103	IncN_1 (repN_1)	*bla*_CTX-M-32_	*aph(3')-Ia*, Δ*ant(3”)-Ia*, *dfrA1*, *lnu*(F), *sul1*	GCA_022591735.1
W49–2	*E. marmotae*	Red deer	Poland	pW49–2-b	55	IncN_1 (repN_1, korA_1, traJ_1)	*bla*_CTX-M-32_	*aph(3”)-Ib*, *aph(6)-Id*, *qnrS1*, *sul2*, *tet*(A)	GCA_022592155.1

*E. marmotae*: *Escherichia marmotae*; *M. wisconsensis*: *Moellerella wisconsensis*; pMLST: plasmid multilocus sequence typing; ni: none identified.

**Figure 2:**
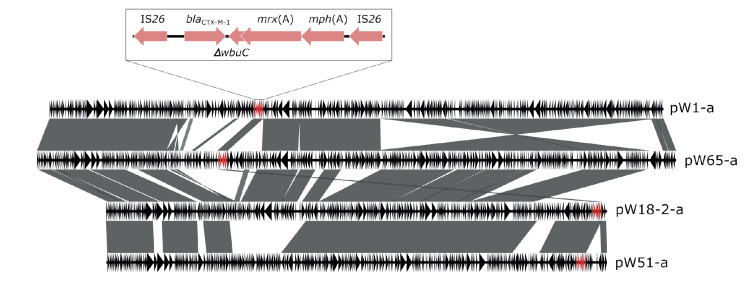
Comparison of *bla*_CTX-M-1_ harbouring megaplasmids identified in *Moellerella wisconsensis* isolates from wild game meat samples, highlighting the transposition units carrying the *bla*_CTX-M-1_ genes, Europe, November 2021 (n = 4 plasmids)

In all instances, the *bla*_CTX-M-1_ genes were found on IS26 flanked transposition units containing *bla*_CTX-M-1_-∆*wbuC*-*mrx*(A)-*mph*(A) (Figure 2). A search with the megaplasmids as queries did not identify any corresponding hits in the bacterial plasmid database PLSDB. *M. wisconsensis* W17–3 and *E. marmotae* W49–2 encoded *bla*_CTX-M-32_ on IncN_1 plasmids. Further, each isolate contained between one and four additional non-*bla*_ESBL_-encoding plasmids (data not shown).

∆*wbuC* is a truncated gene encoding a putative metalloprotein, *mph*(A) encodes macrolide 2'-phosphotransferase I, and *mrx*(A) is an unknown gene involved in macrolide resistance. Grey shades between sequences indicate homologous regions. The figure was produced using EasyFig v. 2.1 (https://mjsull.github.io/Easyfig).

## Discussion

In this study we investigated 111 wild game meat samples obtained locally or imported into Switzerland from the main European game meat-producing countries which are primarily located in Central Europe. We isolated 51 ESBL-producing Enterobacterales including 18 *E. coli*, 32 *M. wisconsensis* and one *E. marmotae*. To the best of our knowledge, this is the first report of MDR ESBL-producing *M. wisconsensis* and *E. marmotae* from wild game meat, although CTX-M-1-producing *M. wisconsensis* was recently described in wild birds in Greece [21], and the isolation of CTX-M-1-producing *E. marmotae* from an Alpine marmot (*Marmota marmotae*) in a Belgian zoo was reported in 2021 [22].

The overall prevalence of ESBL-producing Enterobacterales among the game meat samples in this study was 35%, which amounted to a prevalence of ESBL-producing *E. coli* of 16%. By comparison, for meat derived from domestic food-producing animals in Europe, the prevalence of ESBL-producing *E. coli* in broiler, pig, and bovine meat was 23.4%, 5.5%, and 4.3%, respectively in 2019–2020 [4]. However, with the majority (32/51) of the ESBL-producing Enterobacterales represented by *M. wisconsensis*, our data indicate that using *E. coli* as an indicator may underestimate the true prevalence of ESBL-producing Enterobacterales among samples originating from wildlife and associated environments. *M. wisconsensis* is a rare opportunistic pathogen whose potential role in the dissemination of *bla*_ESBL_ genes in wildlife and in the food chain remains to be to be clarified [21].

Among the samples examined in this work, the occurrence of ESBL-producing Enterobacterales in meat differed between animal species, as well as between countries of origin and suppliers. Meat from red deer showed a higher prevalence (24 of 38 ESBL-producing Enterobacterales) than meat from other animals. Conversely, previous studies published respectively in 2012 and 2018 have shown that faecal carriage of ESBL-producing Enterobacterales in wild red deer is very low in Switzerland (0%) and Poland (0.4%) [23,24]. Thus, the high prevalence of ESBL-producing Enterobacterales found in meat from red deer was unexpected. Similarly, the high proportion of meat containing ESBL-producing Enterobacterales which originated from Austria (6/9), Hungary (5/7), Poland (11/16), and Slovenia (15/22) varies strongly from the low faecal carriage rates of ESBL-producing Enterobacterales in wild game in Austria in 2006 (0%), Central Europe in 2006–2007 (2%), and Poland in 2018 (1.7%) [23,25,26]. Notably, the majority (35/38) of the ESBL positive samples from these countries were provided by the same game processing establishment. By contrast, for meat originating from New Zealand, Germany, and Switzerland the prevalence of ESBL-producing Enterobacterales was very low (0/1, 0/10, and 1/45, respectively). For Germany, these results are supported by a similar study published in 2017, that reported a low prevalence of ESBL-producing *E. coli* (0.4%) in locally sourced and processed wild game meat [7]. Meat samples from these three countries were all supplied by butchers, hunters, or retailers, indicating that practices in handling, processing or distribution of wild game carcasses and meat may have an impact on the prevalence of ESBL-producing Enterobacterales.

The most common ESBL genotype among our isolates was *bla*_CTX-M-1_, which is an animal-associated *bla*_ESBL_ gene found frequently in isolates from domestic animals as well as in wildlife [27]. *E. coli* carrying *bla*_CTX-M-1_ have been recovered from faecal samples collected from red deer in Spain in 2013 and in Switzerland in 2011 [24,28] as well as from Enterobacterales isolated from wild boars in Portugal and Czechia described in 2009 and 2010, respectively [26,29]. While the association of *bla*_CTX-M_ genes with plasmids is well documented for *E. coli* and other Enterobacterales [30], this is to our knowledge, the first description of *bla*_CTX-M-1_ on large (> 270 kb) plasmids in *M. wisconsensis*. Interestingly, the *bla*_CTX-M-1_-carrying megaplasmids (pW18–2-a, pW51-a, pW1-a, and pW65-a, respectively) identified in *M. wisconsensis* were largely homologous, although the isolates had intentionally been selected to be from diverse origins. This suggests that these plasmids are common in CTX-M-1-producing *M. wisconsensis*. In addition, the plasmid sequences had no close match in the PLSDB database, raising the possibility that they are not widespread among other Enterobacterales species. However, since the number of sequenced isolates was limited, further investigations that include a higher number of *M. wisconsensis* are needed to confirm these observations and to assess the molecular features and potential role of these megaplasmids in the dissemination of antimicrobial resistance determinants in wildlife and elsewhere. By contrast, the *bla*_CTX-M-32_ carrying plasmid pW17–3-a identified in *M. wisconsensis* was related to *Proteus mirabilis* plasmid pJPM24 (query coverage: 86%, identity: 99.95%) isolated from chicken faeces in China (NZ_CP053895.1) [31], and the *bla*_CTX-M-32_ harbouring plasmid pW49–2-b from *E. marmotae* was identical with pWE_H_2, a multidrug resistance plasmid which was found among a complex bacterial community taken from wastewater effluent in Sweden MW574948.1 [32], indicating wide dissemination of these ESBL-encoding plasmids.

Based on MLST, no high-risk clones such as *E. coli* ST131 or ST69 were detected [33], however, six of the 18 *E. coli* belonged to STs which have previously been associated with extra-intestinal disease (urinary tract infections) in humans, including *E. coli* ST58, ST68 and ST540 [34]. The *E. coli* STs were to a large extent typical of those found in animals and in humans worldwide, therefore the presence of ESBL genes in isolates from wild game meat may be a consequence of environmental exposure of wildlife to antibiotic residues, resistant bacteria, or resistance genes. AMR Enterobacterales and other microbes from shot animals may be transferred to the carcasses.

Our study has some limitations. The first limitation is that the wild game meat from four of the six the countries investigated (Croatia, Hungary, Poland and Slovenia) came from a single meat processing plant. Hence, it was not possible to distinguish if the occurrence of ESBL-producing Enterobacterales in the game meat from these countries represented carriage acquired in the country of origin by the wild animals, or if it was due to potential cross-contamination at the plant. Second, this study did not include environmental or human samples from the meat processing plant which could provide information on potential cross-contamination among the meat samples, the equipment, the environment, and the workers who handle the meat. Third, while all samples were obtained in Switzerland in November 2021 (some imported and some of local origin), the exact date and geographical location (habitat) of when and where the animals were hunted was unavailable. Therefore, the contribution of environmental exposure (e.g. contact with farmed land) could not be assessed. Finally, due to the considerable differences in sampling numbers, the statistical analysis has limited power. In particular the number of samples from some countries was very small, leading to low precision.

Although in general not consumed raw, game meat represents a risk of exposure to ESBL-producing Enterobacterales through direct contact with the raw meat, through consumption of undercooked meat, and through cross-contamination or re-contamination processes between raw meat and foods that are consumed raw. The potential transfer of ESBL-producing Enterobacterales to humans constitutes a public health risk, as such bacteria may transfer antimicrobial-resistance genes to pathogenic bacteria within the human gut.

## Conclusions

This study revealed an occurrence of ESBL-producing Enterobacterales in about a third of wild game meat samples from central European countries. Our data further indicate that the prevalence of ESBL-producing Enterobacterales in wildlife may be higher than previously anticipated, and that microbial contamination and cross-contamination events associated with harvesting of wild game carcasses, as well as cross-contamination within game meat processing establishments may contribute to a high contamination of the final meat product.

Raised awareness for adequate handling of wild game meat should be considered to mitigate the risk of transmission of antibiotic resistant Enterobacterales to the consumer.
